# Phylogeography and conservation gaps of *Musa balbisiana* Colla genetic diversity revealed by microsatellite markers

**DOI:** 10.1007/s10722-022-01389-4

**Published:** 2022-05-07

**Authors:** Arne Mertens, Yves Bawin, Samuel Vanden Abeele, Simon Kallow, Rony Swennen, Dang Toan Vu, Tuong Dang Vu, Ho Thi Minh, Bart Panis, Filip Vandelook, Steven B. Janssens

**Affiliations:** 1 Department of Biosystems, Laboratory of Tropical Crop Improvement, KU Leuven, Leuven, Belgium; 2 Meise Botanic Garden, Meise, Belgium; 3 Department of Biology, KU Leuven, Leuven, Belgium; 4 Royal Botanic Gardens Kew, Millennium Seed Bank, Ardingly, UK; 5 International Institute of Tropical Agriculture, Kampala, Uganda; 6 Research Planning and International Cooperation Department, Plant Resources Center, Hanoi, Vietnam; 7 Faculty of Agronomy, Vietnam National University of Agriculture, Hanoi, Vietnam; 8 Bioversity International, Leuven, Belgium

**Keywords:** Banana, Crop wild relatives, Ex situ conservation, Genetic diversity, *Musa balbisiana*, SSR

## Abstract

Collection and storage of crop wild relative (CWR) germplasm is crucial for preserving species genetic diversity and crop improvement. Nevertheless, much of the genetic variation of CWRs is absent in ex situ collections and detailed passport data are often lacking. Here, we focussed on *Musa balbisiana,* one of the two main progenitor species of many banana cultivars. We investigated the genetic structure of *M. balbisiana* across its distribution range using microsatellite markers. Accessions stored at the International *Musa* Germplasm Transit Centre (ITC) ex situ collection were compared with plant material collected from multiple countries and home gardens from Vietnam. Genetic structure analyses revealed that accessions could be divided into three main clusters. Vietnamese and Chinese populations were assigned to a first and second cluster respectively. A third cluster consisted of ITC and home garden accessions. Samples from Papua New Guinea were allocated to the cluster with Chinese populations but were assigned to a separate fourth cluster if the number of allowed clusters was set higher. Only one ITC accession grouped with native *M. balbisiana* populations and one group of ITC accessions was nearly genetically identical to home garden samples. This questioned their wild status, including accessions used as reference for wild *M. balbisiana*. Moreover, most ITC accessions and home garden samples were genetically distinct from wild populations. Our results highlight that additional germplasm should be collected from the native distribution range, especially from Northeast India, Myanmar, China, and the Philippines and stored for ex situ conservation at the ITC. The lack of passport data for many *M. balbisiana* accessions also complicates the interpretation of genetic information in relation to cultivation and historical dispersal routes.

**Supplementary Information:**

The online version contains supplementary material available at 10.1007/s10722-022-01389-4.

## Introduction

Crop wild relatives (CWRs) are wild species that are closely related to their associated crop and are a source of genetic diversity for crop improvement. They potentially contain new key alleles against environmental stressors or are desirable for the modification of quantitative and qualitative crop traits such as yield, taste, and shape (Hajjar and Hodgkin 2007; Dempewolf et al. 2017). To safeguard the role of CWRs for future plant breeding, it is very important to collect, identify, protect and use this genetic variation present in CWRs in both in situ and ex situ conservation (Heywood et al. 2007; Maxted and Kell 2009; Castañeda-Álvarez et al. 2016).

In the last decades, several efforts have been undertaken for conservation of CWRs ex situ as there is a growing awareness of their importance for crop improvement and food security (Hajjar and Hodgkin 2007; McCouch 2013; Dempewolf et al. 2017). However, CWRs are still poorly represented in gene banks (Castañeda-Álvarez et al. 2016; Khoury et al. 2021; Mertens et al. 2021b). For example, over 4.1 million accessions are listed in the Genesys global database for crop diversity conserved in gene banks (Genesys 2021) and only 14% of them are considered wild in origin. Based on the European Search Catalogue for Plant Genetic Resources (EURISCO), Ford-Lloyd et al. (2011) demonstrated that only 6% of European CWR species are conserved in ex situ collections. Moreover, many of these species in collections are only represented by a single specimen that is further clonally propagated. This extremely narrow collection basis has several drawbacks: (i) only a subset of the genetic variation is captured from the population where it was sampled from; (ii) there is a risk of genetic drift during regeneration, especially when the original sampling size is low; (iii) when conserved in vitro*,* somaclonal variation may arise, further differentiating the germplasm accessions from the original wild populations (Krishna et al. 2016); and (iv) ex situ collections are not prone to the same biological (species interactions) and abiotic (climate) processes compared to wild populations, therefore withholding their adaptation to gradual changes in environmental conditions (Meilleur and Hodgkin 2004; Heywood 2016). Moreover, not all species can be conserved ex situ due to either specific ecosystem interactions or limitations in seed storage such as seeds with recalcitrant or intermediate storage classification (Rasmussen et al. 2015).

Besides the need to expand the total number of CWRs in gene banks, the metadata associated with stored CWRs is of vital importance for further use. Although guidelines have been developed for the collection and management of CWR data (e.g. descriptors for uploading passport data to EURISCO or Genesys), such information is often lacking. Moreover, there is currently no standardised format or global portal to access data that are specifically gathered on CWRs (Engels and Thormann 2020).

Bananas (Musaceae) are one of the worlds’ most important staple foods. With over 150 million tonnes being produced annually, they contribute to the income and diets of hundreds of millions of people (FAO 2018, 2019). There are 83 wild banana species (*Musa* L.) according to the World Checklist for Selected Plant families (WCSP 2021), and over 1,000 varieties have been described (Daniells et al. 2001; Ploetz et al. 2007; Perrier et al. 2011; Ruas et al. 2017). Worldwide, a total of 31 field and in vitro collections conserve 6,772 banana accessions (Ruas et al. 2017), of which 1617 accessions are stored at the International *Musa* Germplasm Transit Centre (ITC), with 1100 accessions being duplicated and conserved cryogenically (Van den houwe et al. 2020). To date, the ITC has the largest collection of *Musa* germplasm in the world with the long term security of the banana gene pool as main goal, while also globally providing pest- and disease-free germplasm (Van den houwe et al. 2020). Within the ITC, most acquisitions (84%) are cultivated bananas. Moreover, the 16% wild accessions at the ITC comprise 34 Musaceae species, yet these are often represented by only one clonally reproduced genotype. Next to in vitro collections, many institutes hold banana seed collections, such as the Plant Resources Center in Vietnam and the Millennium Seed Bank in Great Britain (Kallow et al. 2022). Banana seed storage can be a cost-efficient complementary method for the long-term conservation and distribution of banana genetic resources (Li and Pritchard 2009), but many issues remain unresolved regarding their collection, seed banking, and germination (Brown et al. 2017; Kallow et al. 2020b, a; Panis et al. 2020).

Germplasm from wild or cultivated banana material can be requested from gene banks with information coming from the *Musa* Germplasm Information System (MGIS) (Ruas et al. 2017). Accessions should typically come with passport metadata that includes information on taxonomy, ecology, geography, and ethnobotanical uses. Such data are also helpful to develop breeding programmes and strategies for collecting additional germplasm. Passport data are required to determine what part of the gene pool is insufficiently conserved and to pinpoint favourable geographical regions for additional germplasm collection (Meyer 2015; Weise et al. 2020). However, this information is often missing, unavailable online, or unknown (Dempewolf et al. 2017). For example, georeferenced sampling locations of germplasm accessions are important to carry out a gap analysis to identify which region should be explored or prioritized for future collecting.

Northern Indo-Burma was recently revealed as the region of origin of the banana family (Musaceae) (Janssens et al. 2016) and this region was also marked with a high climatic suitability for many banana species (Mertens et al. 2021b). However, species specific assessments of genetic variation on a large geographic scale have rarely been assessed in this family.

In this study, we focussed on *Musa balbisiana* Colla, the single progenitor of the BBB cultivar group as well as the donor of the “B genome” to hybrid cultivars with *M. acuminata* Colla (“A genome”) belonging to the AB, ABB, AAB, AABB, and ABBB groups (Simmonds and Shepherd 1955). *Musa balbisiana* is a wild diploid species native to (sub)-tropical rainforests ranging from Northeast India to South China and northern Vietnam. Although the species is also present in Taiwan, the Ryukyu islands (Japan), Indonesia, Malaysia, the Philippines, and Papua New Guinea (PNG), these occurrences have been attributed to human-mediated introductions (De Langhe et al. 2015). While *M. balbisiana* is not parthenocarpic and has seedy fruits, it is regularly cultivated and many parts of the plants are used for food, fodder, fibre, wrapping material or medicine (Kennedy 2009). After being introduced into regions with suitable climatic conditions, *M. balbisiana* establishes vigorous populations, which are typically called “feral”. Although the Philippines are often considered as part of the native distribution area of *M. balbisiana* because of its widespread presence across multiple Philippine provinces (Sotto and Rabara 2000), De Langhe et al. (2015) suggested that *M. balbisiana* accessions were introduced, based on the small variation in local AAB cultivar subgroups that developed there since the start of the cultivation of edible *M. acuminata* Colla (AA group). In addition, the absence of *M. balbisiana* in other parts of Marine Southeast Asia and its scattered distribution in PNG may indicate non-natural introductions in the Philippines, but it is no conclusive evidence (De Langhe et al. 2015).

Up to now, few studies have focussed on the genetic diversity present among wild *M. balbisiana* populations within the native distribution range (Ge et al. 2005; Uma et al. 2006; Wang et al. 2007). Often, genetic research is done on *M. balbisiana* material obtained from ex situ collections (Ude et al. 2002; Youssef et al. 2011; Bawin et al. 2019), grown outside the native distribution range (Ahmad et al. 2014), or with cultivated material and hybrids (Doloiras-Laraño et al. 2018). Moreover, the results of those studies are difficult to compare, as they use different methods and genetic markers (e.g. AFLP, SSRs, RAPD). More recently, two studies used a set of 18 polymorphic SSR markers to interlink genetic variation in wild *M. balbisiana* accessions with that in ex situ collections. Bawin et al. (2019) compared the genetic diversity of seeds from wild *M. balbisiana* populations from Yunnan (China) with those from ex situ seed collections, whereas Kallow et al. (2021) compared seed collections to source populations of *M. balbisiana*. In the present study, we investigated the genetic structure of *M. balbisiana* in its complete distribution range (native + feral) in Southeast Asia and Melanesia. With this approach we aim to (i) identify areas in Southeast Asia that require additional collecting; (ii) investigate the extent of the genetic variation in *M. balbisiana* accessions that are currently stored and maintained in the ITC gene bank; and (iii) examine the importance of passport data for linking genetic data with the putative interpretation of human interference and dispersal routes.

## Methods

### Taxon sampling

**Fig. 1:**
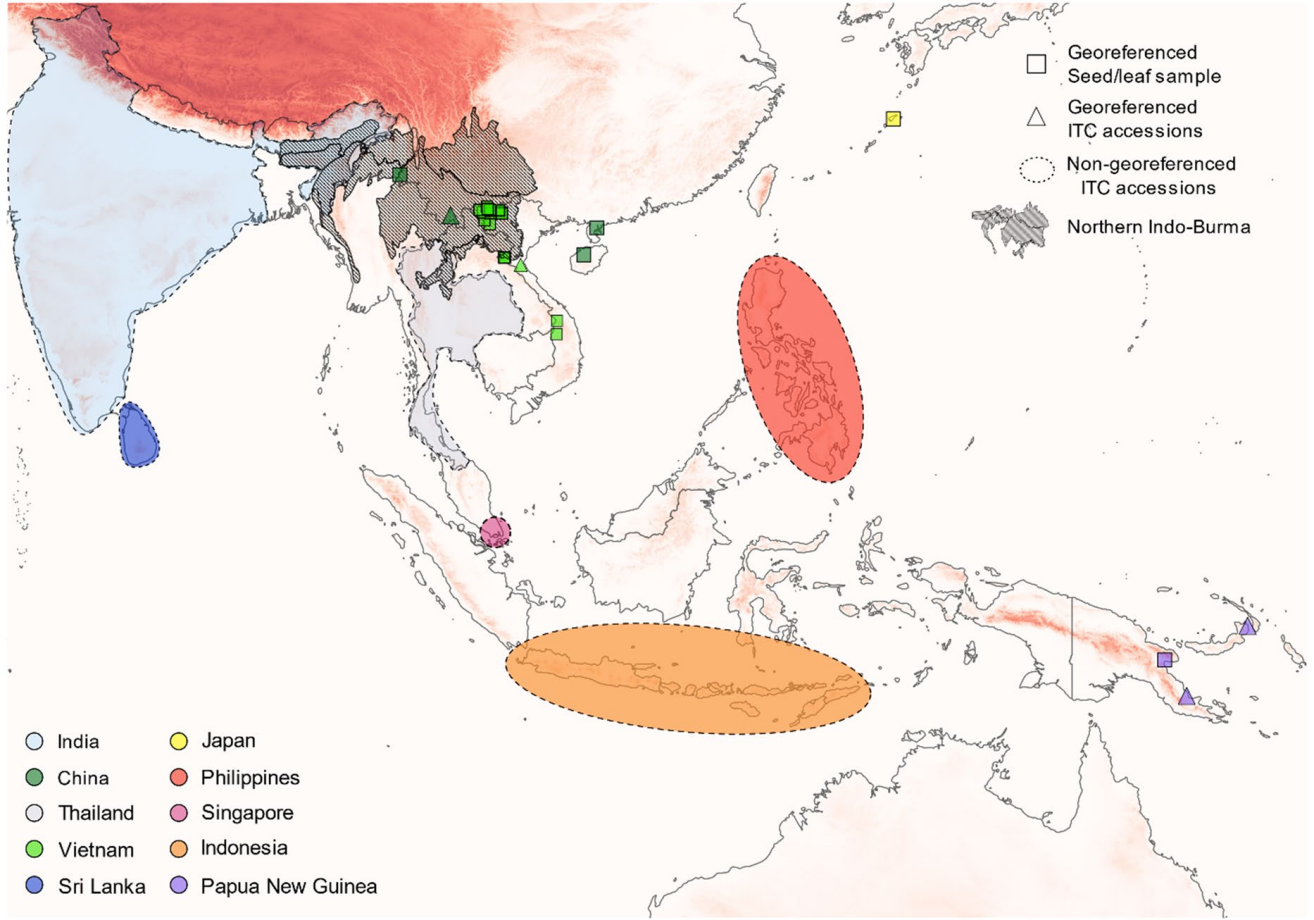
Locations of *M. balbisiana* accessions used in this study. Triangles indicate georeferenced accesions from the ITC collection, while squares marked georeferenced samples collected from leaves or seed collections. Areas encircled with a dashed line indicate regions where non-georeferenced ITC collections were presumably collected. Symbol colours specify the country of origin. The dark shaded area represents northern Indo-Burma, the presumed region of origin of the banana family. The red shaded background depicts elevation. (Color figure online)

*Musa balbisiana* samples from different countries were acquired from three different sources (Supplementary Information – Table 1). Firstly, SSR data of *Musa balbisiana* were retrieved from two previous studies (Bawin et al. 2019; Mertens et al. 2021a). Secondly, 28 different germplasm accessions of *M. balbisiana* from the ITC (one sample per accession) originating from the Philippines, Indonesia, India, PNG, China, and Thailand were included (Fig. 1). Thirdly, to trace the origin of plants grown in home gardens in Vietnam, leaf material from six *M. balbisiana* accessions grown in home gardens in South-Central Vietnam and one from a home garden in North Vietnam were selected. Single or multiple plants in gardens or next to houses from villagers were considered as home garden samples.

The dataset included a total of 372 samples from four material types: 225 samples from 17 wild populations of *Musa balbisiana*, 21 samples from six home gardens, 98 samples retrieved from eight seed collections, and 28 accessions of *M. balbisiana* from the in vitro collection of the ITC.

The accuracy of the passport data regarding the original sampling location of the 28 ITC accessions varied substantially. Five accessions had accurate passport data with sampling coordinates, whereas four others had a detailed description of their sampling region (e.g. name of the province within the country of origin) without coordinates. Eleven samples only had information on their country of origin, and eight samples had no data on their sampling origin (Supplementary Information – Table 1).

### Genotyping and sequencing

#### DNA extraction

Leaf material from field missions and ITC germplasm was dried using silica gel and DNA was subsequently isolated using a modified cetyltrimethylammonium bromide (CTAB) extraction protocol (Doyle and Doyle 1987). For seed collections, embryos were first excised using a sterile scalpel and were then added to a 10 µl CTAB solution for DNA isolation using the same protocol.

#### SSR genotyping

We used the same set of 18 microsatellite markers arranged in four multiplexes as described in Bawin et al. (2019) to compare the ITC samples with those used in Bawin et al. (2019) and Mertens et al. (2021a). Markers were coupled to universal primer sequences (Schuelke 2000) and DNA fragments were amplified with PCR using the Type-it Microsatellite PCR Kit (Qiagen, Venlo, The Netherlands). We added 1 µl of diluted PCR sample to a 12 µl HiDi formamide solution mixed with 0.4 µl of MapMarker 500 labelled with DY-632 (Eurogentec, Seraing, Belgium), and 1.5 µl of this product was then genotyped on an ABI 3730 sequencer (Applied Biosystems, Foster City, California, USA) at the Université Libre de Bruxelles (ULB, Belgium). PCR product of reruns and of more recently sampled material (20 µl per sample) was sent to Macrogen (Macrogen Europe, Amsterdam, The Netherlands) for genotyping on an ABI 3730xl system. To deal with potential differences in fragment sizes between the two sequencers, a minimum of ten samples per run were analysed in duplicate. Raw data were scored in Geneious Prime 2021.1.1 using the 3^rd^ order least square sizing method (Kearse et al. 2012). All data obtained from the study of Bawin et al. (2019) were rescored using the same sizing method. Samples showing ambiguous genotyping patterns were genotyped twice to avoid erroneous scoring. Samples with more than 10% missing data were excluded from the analyses.

#### Sanger sequencing

We additionally screened each ITC accession and three random samples of each population with one nuclear marker (ITS) and three chloroplast markers (*rps16, trnL-F, atpB-rbcL*) using the PCR protocols described by White et al. (1990), Oxelman et al. (1997), Taberlet et al. (1991), and Chiang et al. (1998) respectively. PCR samples were sent to Macrogen for sequencing (Macrogen Europe, Amsterdam, The Netherlands). Forward and reverse sequences were checked for quality and assembled in Geneious Prime (Kearse et al. 2012). All *M. balbisiana* sequences were subsequently aligned using the MAFFT Alignment tool (Katoh and Standley 2013) implemented in Geneious with the E-INS-I algorithm, a scoring matrix of 100PAM / K = 2, and a gap open penalty of 1.3. To identify potential hybrids in the ITC collection, Sanger sequences were blasted against the NCBI nucleotide database (NCBI Resource Coordinators 2018). Sequences were additionally uploaded to NCBI GenBank (Accession numbers OK648712-OK649230).

### Genetic analyses

As this study aimed to identify areas in the putative native distribution area of *M. balbisiana* that need additional sampling for ex situ conservation, accessions obtained from outside Asia and PNG (as indicated by accession names or donor institute) or those marked as hybrids in the *Musa* germplasm information system (MGIS) were excluded from the analyses of genetic structure (Ruas et al. 2017).

#### Allelic diversity

For each accession, the percentage of polymorphic loci (*%P*), the average number of different alleles (*Na*), the average number of unique (private) alleles (*Np*), and the number of locally common alleles (frequency ≥ 5% in a population) found in less than 25% of all assessed accessions (*Lcomm*) were calculated using the “allele frequency data parameters” menu in the GenAlEx 6.51 Excel plugin (Peakall and Smouse 2012).

#### Genetic structure

We calculated a genetic distance matrix based on the codominant genotypic distances and ran a Principal Coordinate Analysis (PCoA) in GenAlEx using the “Distance-Based” menu options. For this analysis, all individuals from each population were used and the data were presented both at the population level (one point per population) and at the individual level (each individual is plotted). As ITC accessions cannot be seen as one population due to their dispersed origin, we refrained from performing an analysis of molecular variance (AMOVA). In another analysis, a similar plot was made including six accessions of unknown origin to see whether their putative origin could be traced back (ITC0080, ITC0211, ITC0212, ITC0246, ITC0247, ITC0271).

Next to PCoA, we used Bayesian clustering in the STRUCTURE software (Pritchard et al. 2000) to infer genetic structure in our sampling. To find the optimal number of clusters (*K*), we ran an admixture model to allow samples to be assigned to one or multiple clusters. Unequal sampling of populations may lead to inaccurate estimation of *K* (Wang 2017). To compensate for the presumably low number of accessions per population present in the ITC collection, we reduced at random the number of samples of remaining populations to five. In order to obtain accurate results with biased sampling using STRUCTURE, we used a model with uncorrelated allele frequencies, a separate alpha (a prior of individual ancestry) for each population, and an initial alpha of 1/*K* (Wang 2017). To infer this *K* value, we first carried out 10 preliminary runs using a dependent model and correlated allele-frequencies with an initial alpha of 1. For each run, 100,000 Markov Chain Monte Carlo iterations were sampled after a burn-in of 25,000 iterations. To determine the optimal number of clusters, we considered both the Δ*K*/*K* and the log posterior probability of the replicates over each *K* (Evanno et al. 2005) with the online web server of StructureSelector (Li and Liu 2018). This *K* was then used to optimize the assignment of individuals by running a new admixture model with independent allele frequencies and an initial alpha value of 1/*K*. Bar plots with the assignment probabilities obtained using STRUCTURE were subsequently made with the CLUMPAK software integrated in StructureSelector (Kopelman et al. 2015; Li and Liu 2018).

## Results

### DNA barcoding

Screening of both nuclear (ITS) and chloroplast (*rps16, trnL-F, atpB-rbcL*) markers revealed very little sequence variation between accessions. BLAST resulted in sequence similarities with *M. balbisiana* ranging from 99.5–100% for all markers. Nonetheless, based on the chloroplast markers, five accessions (ITC0094, ITC0545, ITC1788, ITC1789, and ITC1823) were found to be potential hybrids with *M. acuminata* (> 99.1% sequence similarity).

### Allelic diversity

For the subsequent analyses, the dataset was reduced to accessions presumed to be diploids of *M. balbisiana* of which the country of origin was known, thus excluding accessions that showed high similarity of chloroplast sequences with *M. acuminata*. For accession ITC0248 “Singapuri”, the country of origin was inferred to be Singapore. From all assessed microsatellite loci, BB_CT-33, BB_GAA-4, BB_CT-6, and BB_CT-8 were the most informative. While five loci were uninformative (monomorphic) for the samples included in this study (Mbg02, Mbg04, BB_AAC-3, BB_CT-7, and Mbg01), they were retained for all analyses in order to be able to compare results with the studies of Bawin et al. (2019) and Mertens et al. (2021a). The proportion of polymorphic loci (*%P*) and average number of different alleles (*Na*) ranged from 0 to 66.67 and 1.000 to 2.167 respectively (Table 1). The proportion of polymorphic loci was lowest in the seed collection of Amami (Japan) followed by Vietnamese populations VNM-N8 and VNM-S1, and the seed collection of PNG, whereas it was highest in the Central Vietnam population VNM-C2. No polymorphic loci were found in the seed collection of Amami and the ITC accessions from PNG. The highest number of private alleles was found in populations of Central Vietnam (VNM-C1, VNM-C2) and a home garden in South Vietnam (VNM-S3). Apart from VNM-S3, *M. balbisiana* from home gardens in Vietnam and five accessions of the ITC, including the ones sampled from Vietnam (ITC1681, ITC1687), had no locally common alleles. Excluding VNM-S3, no home garden or ITC accession had private alleles.

**Table 1:** Sample size, percentage of polymorphic loci (*%P*), average number of different alleles (*Na*), proportion of private alleles (*P*), proportion of locally common alleles with a frequency lower than 25% (*Lcomm*) of populations and accessions used in this study.

Population	Sample size	*%p*	*Na*	*P*	*Lcomm*	Status
VNM-N1	16	22.22	1.222	0.000	0.000	Population
VNM-N2	15	38.89	1.667	0.000	0.222	Population
VNM-N3	11	16.67	1.167	0.000	0.000	Population
VNM-N4	15	50.00	1.611	0.056	0.111	Population
VNM-N5	13	50.00	1.722	0.000	0.278	Population
VNM-N6	13	44.44	1.611	0.000	0.222	Population
VNM-N7	13	22.22	1.222	0.056	0.056	Population
VNM-N8	11	11.11	1.111	0.000	0.056	Population
VNM-N9	15	27.78	1.278	0.000	0.000	Home garden
VNM-N10	15	27.78	1.278	0.000	0.222	Population
VNM-N11	14	44.44	1.944	0.056	0.333	Population
VNM-N12	15	50.00	1.889	0.000	0.389	Population
VNM-N13	15	38.89	1.722	0.000	0.278	Population
VNM-N14	4	27.78	1.278	0.000	0.056	Population
VNM-C1	15	55.56	1.944	0.111	0.389	Population
VNM-C2	15	66.67	2.167	0.167	0.389	Population
VNM-S1	15	11.11	1.111	0.000	0.222	Population
VNM-S2	10	16.67	1.167	0.056	0.111	Population
CHN-W1	12	61.11	1.833	0.000	0.389	Population
CHN-W2	14	50.00	1.778	0.000	0.444	Population
CHN-W3	14	33.33	1.333	0.056	0.167	Population
CHN-W4	8	55.56	1.833	0.000	0.444	Population
CHN-S	5	44.44	1.611	0.056	0.111	Population
CHN-Hainan	15	44.44	1.667	0.000	0.278	Population
JPN-Amami	14	0.00	1.000	0.000	0.056	Population
PNG	15	11.11	1.111	0.056	0.056	Population
VNM-S3	1	38.89	1.389	0.278	0.111	Home garden
VNM-S5	1	33.33	1.333	0.000	0.000	Home garden
VNM-S6	1	33.33	1.333	0.000	0.000	Home garden
VNM-S7	1	27.78	1.278	0.000	0.000	Home garden
VNM-S8	1	33.33	1.333	0.000	0.000	Home garden
VNM-S9	1	27.78	1.278	0.000	0.000	Home garden
ITC0248—SGP	1	44.44	1.444	0.000	0.389	ITC
ITC0564—PHL	1	22.22	1.167	0.000	0.167	ITC
ITC0565—PHL	1	33.33	1.333	0.000	0.111	ITC
ITC0626—PNG	1	0.00	1.000	0.000	0.056	ITC
ITC1016—PNG	1	0.00	1.000	0.000	0.056	ITC
ITC1120—THA	1	33.33	1.333	0.000	0.000	ITC
ITC1527—CHN-SW	1	22.22	1.222	0.000	0.222	ITC
ITC1587—IDN	1	33.33	1.333	0.000	0.000	ITC
ITC1588—IND	1	44.44	1.444	0.000	0.389	ITC
ITC1681—VNM	1	33.33	1.333	0.000	0.000	ITC
ITC1687—VNM	1	33.33	1.333	0.000	0.000	ITC
ITC1780—PHL	1	33.33	1.333	0.000	0.111	ITC
ITC1787—PHL	1	16.67	1.167	0.000	0.111	ITC
ITC1790—PHL	1	22.22	1.222	0.000	0.111	ITC
ITC1875—IDN	1	22.22	1.222	0.000	0.000	ITC

*VNM* Vietnam, *CHN* China, *JPN* Japan, *PNG* Papua New Guinea, *SGP* Singapore, *PHL* the Philippines, *THA* Thailand, *IDN* Indonesia, *IND* India

### Genetic structure

#### Principal coordinate analysis

**Fig. 2:**
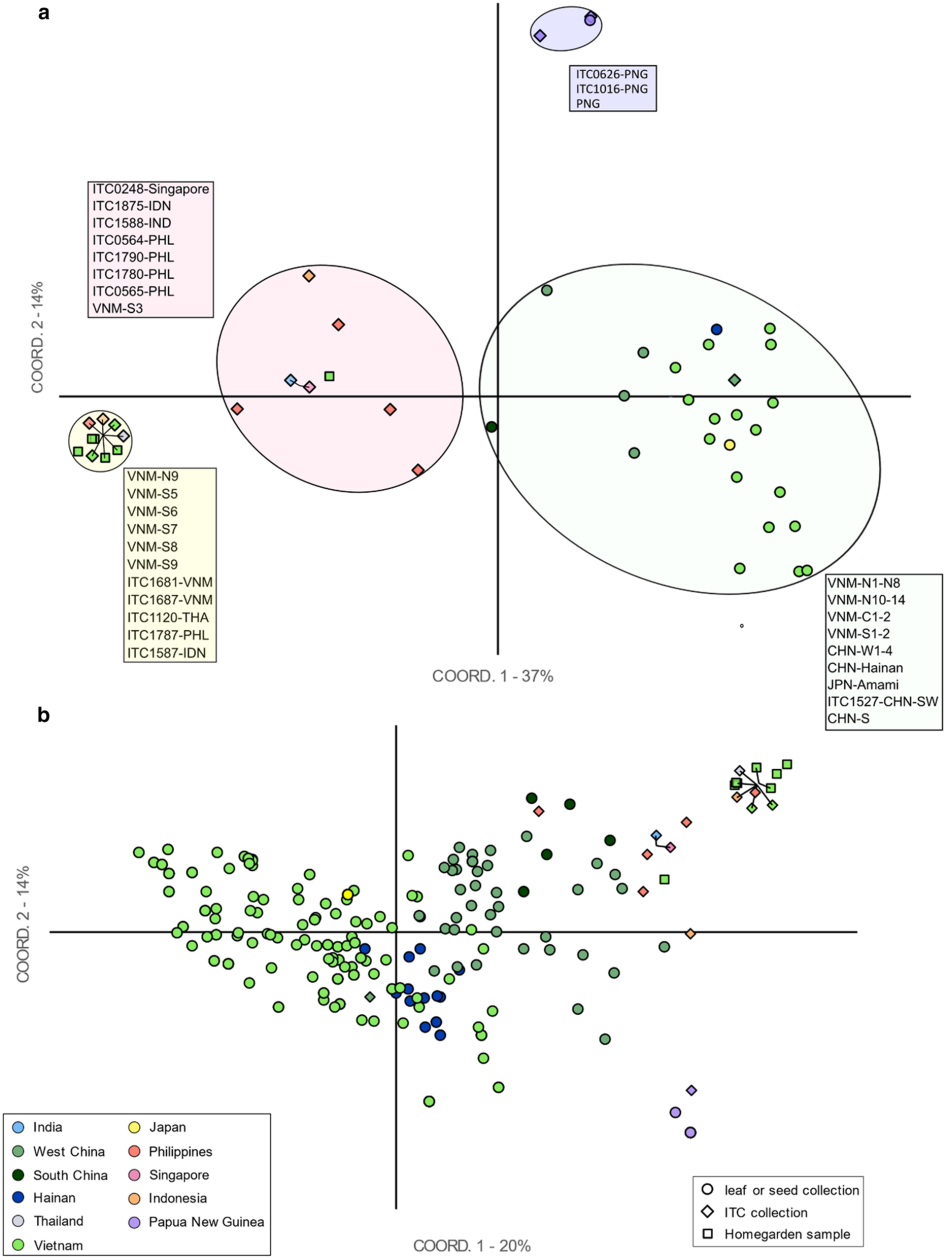
Principal Coordinate Analysis (PCoA) of *M. balbisiana* samples of known origin based on codominant genotypic distance of the reduced SSR dataset. Symbol colours indicate regions of origin and symbol shape defines sample type. Accessions connected with a line indicate that they were genetically identical for the assessed SSRs. **a** Each population/accession is represented by one data point. Four groups are indicated and corresponding accessions or population names are represented in the same colour. **b** Each sample/accession is represented by one data point

In the Principal Coordinate Analysis for all populations and accessions with a labelled country of origin, the first two axes explained 51% of the observed variance (37% and 14% respectively). Five main patterns were observed in the PCoA graph with the populations from Vietnam, China, PNG, and Japan plotted together with the distinct ITC and home garden accessions (Fig. 2a). First, populations from China and Vietnam (green: native distribution group) were clearly distinct from home garden samples from northern and southern Vietnam, as well as most of the ITC accessions studied. Second, ITC accessions and the seed collection of PNG (purple: PNG group) were clearly distinct from other accessions. Third, ITC1587 from Indonesia closely grouped together with an accession from Thailand (ITC1120), the Philippines (ITC1787), two ITC accessions from Vietnam (ITC1687, ITC1681), and most of the *M. balbisiana* sampled from home gardens in Vietnam (VNM-S5-8 and VNM-N9) (yellow group). Fourth, another group of ITC accessions from various countries of origin and one home garden sample from Vietnam (VNM-S3) were genetically similar (pink group). Fifth, only one accessions from ITC (ITC1527) from Xishuangbanna (China) grouped among accessions from the native distribution area. The same patterns were found in the PCoA graph where individual samples from populations were plotted (Fig. 2b). The two first axes explained 20% and 14% of the observed variance respectively. Some accessions from Chinese populations (mainly from CHN-S from Guangdong and CHN-W1 from Yunnan) were genetically relatively close to a set of ITC accessions and VNM-S3 (pink circle in Fig. 2a).

**Fig. 3:**
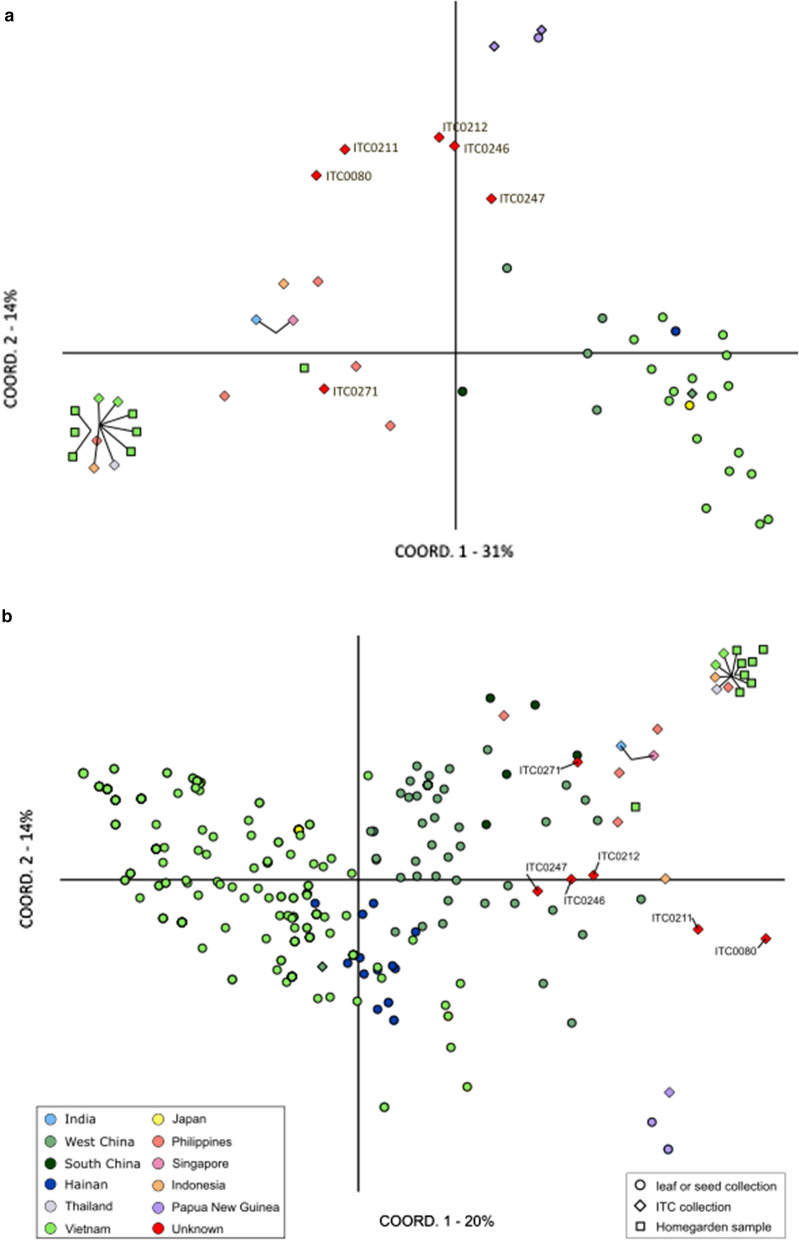
Principal Coordinate Analysis (PCoA) of *M. balbisiana* accessions including accessions from unknown geographic origin. Symbol colours indicate known regions of origin and symbol shape defines sample type. Accessions connected with a line indicate that they were genetically identical for the assessed SSRs. **a** Each population/accession is represented by one data point. **b** Each sample/accession is represented by one data point

The PCoA including the six accessions of unknown origin showed that, apart from one accession (ITC0271), all these accessions grouped together and seemed genetically relatively distinct from other accessions when assessed at the population level (Fig. 3a). However, when screening them at the sample level (Fig. 3b), three accessions of unknown origin (ITC0212, ITC0246 “Cameroun”, ITC0247 “Honduras”) are genetically close to some samples of West China, while ITC0080 and ITC0211 are genetically more distant from other samples.

#### STRUCTURE analyses

**Fig. 4:**
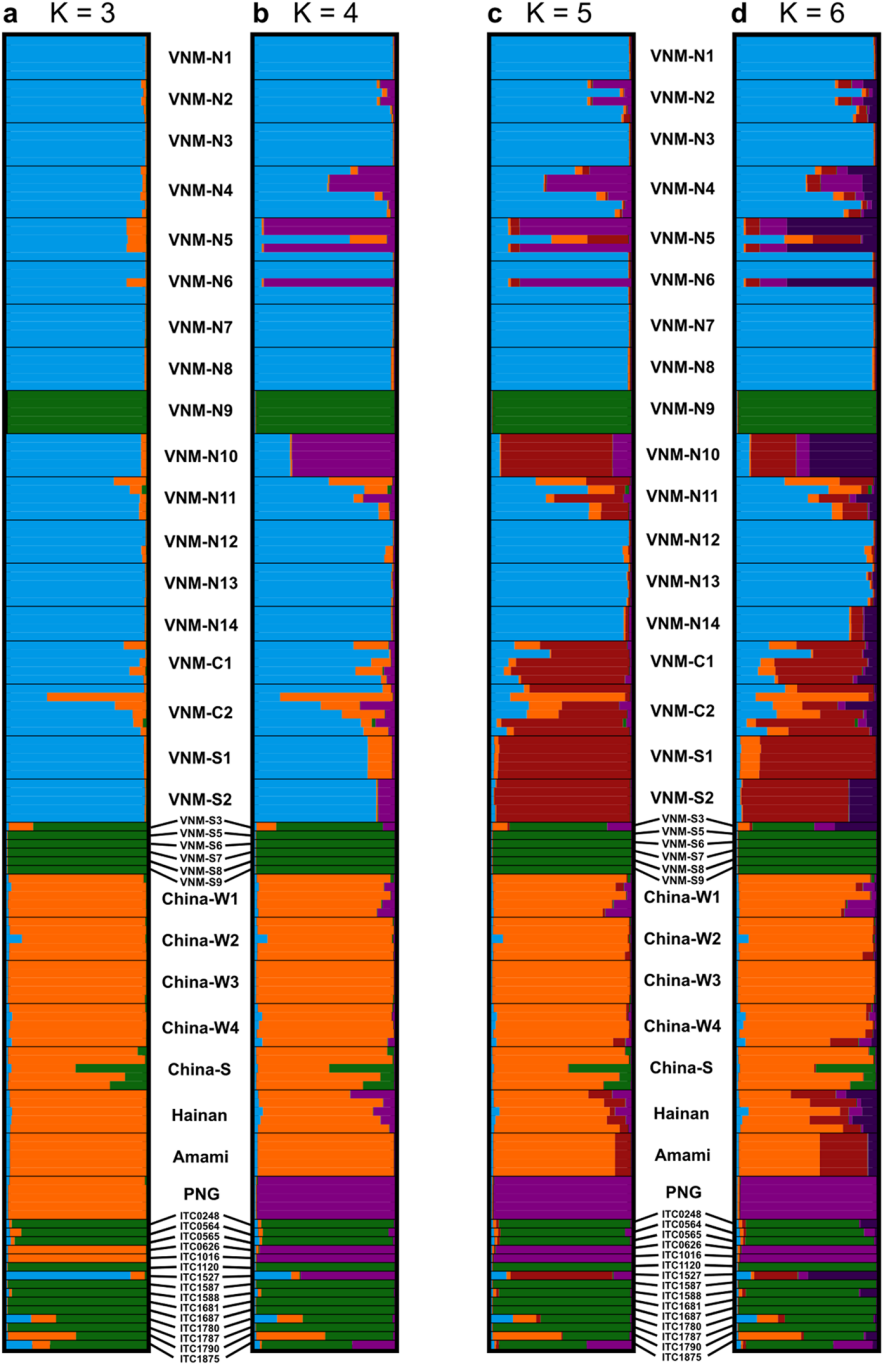
STRUCTURE bar plots based on 18 SSR markers for a subsampled dataset generated using CLUMPAK with the number of genetic groups ranging from *K* = 3 to *K* = 6. Each horizontal line represents one individual and the assignment probabilities were based on all 10 iterations rather than on the run with the highest log likelihood

In the preliminary run using STRUCTURE with correlated allele frequencies and an initial alpha of 1, the Δ*K*/*K* was highest for *K* = 3 (13.42) and second highest for *K* = 6 (4.39). The logarithm of the posterior probability of the replicates over each *K* suggested an optimal *K* of 19, but the slope of the curve sharply decreased and the standard deviation started to increase for a *K* larger than 6 (Supplementary Information – Fig. 1). For this reason, *K* = 6 was used to set an initial alpha of 0.167 (1/6) in the subsequent clustering runs with uncorrelated allele frequencies to obtain a better cluster assignment for each individual. Because an ‘optimal *K*’ can be quite ambiguous and because Δ*K*/*K* clearly showed *K* = 3 as the preferred number of genetic groups, we here report STRUCTURE plots ranging from *K* = 3 to *K* = 6 (Fig. 4a–d).

When *K* was set to three (Fig. 4a), three well-defined groups were delineated. All Vietnamese populations clustered together in a first group together with the ITC accession originating from Southwest China (Blue), showing little admixture. All other accessions sampled in China grouped together in a second cluster (orange), together with the seed accessions of Amami (Japan), the ITC accessions from PNG, and the seed collection of PNG. A third cluster (green) included all remaining ITC accessions, the home garden accessions from South Vietnam and VNM-N9 from northern Vietnam. Some individuals of VNM-C2 in the first cluster and of CHN-S in the second cluster displayed admixture with the second and third cluster, respectively. One ITC sample (ITC1790) from the Philippines had a hybrid genotype of the second and the third group. When K was set to 4 (Fig. 4b), the accessions from PNG were assigned to a separate fourth cluster (purple) that also contained some individuals from VNM-N4, VNM-N5, and VNM-N6. Accessions from VNM-N10 and the ITC accession from SW China also showed affinity with this genetic group, but showed high levels of admixture. This is all in accordance with the PCoA (Fig. 2a). When *K* was further increased to 5 or 6 (Fig. 4c and d respectively), we observed some additional sub-structure in Vietnamese populations, mainly in Central and southern Vietnam. Although individuals were more poorly assigned to clusters at higher *K*-values (data not shown), in general, the main clusters as observed at *K* = 4 remained clear, with additional sub-structure in some Vietnamese populations.

## Discussion

### Genetic structure of *M. balbisiana* accessions in Southeast Asia

In order to place our observed genetic structure within the current knowledge on the history of *M. balbisiana* movement, it is important to take into account the origin of the species and current knowledge on the introduction of this species across insular Southeast Asia. Our results provide evidence for the genetic structuring of the accessions in four groups: (i) wild Chinese populations, (ii) wild Vietnamese populations, (iii) most ITC and home garden accessions from Vietnam, and (iv) accessions from PNG.

#### Chinese and Vietnamese populations

A previous study of Mertens et al. (2021a) demonstrated that Chinese and northern Vietnamese populations contained high genetic diversity which is important to conserve while additional sampling in Central Vietnam was recommended based on the high genetic diversity and number of unique alleles in the two sampled populations. Vietnamese populations could be distinguished from Chinese populations, with even further substructuring of Vietnamese populations at higher *K*-values. In the current study, the same pattern was observed when accessions from other regions were included in the dataset. The seed collection of Amami (Japan) and ITC1527 from Southwest China clustered with Chinese and Vietnamese populations respectively. A Chinese introduction of *M. balbisiana* in the 16th century in Japan has already been suggested, bringing great economic benefits to the Ryukyu islands (Kennedy 2009; De Langhe et al. 2015). The lack of genetic variation in the 15 samples from Amami, which is part of the Ryukyu islands, supports the assumption of a narrow genetic basis in this region. Furthermore, ITC1527 from Xishuangbanna in southern Yunnan grouped with populations of Vietnam rather than with other Chinese populations. Xishuangbanna is geographically close to northern Vietnam and the composition of the tropical seasonal rainforests of Xishuangbanna and the forests in north-western Vietnam are floristically similar, possibly connecting *M. balbisiana* populations in China and northern Vietnam (Lü et al. 2010).

#### ITC accessions and home gardens of Vietnam

Most other ITC and Vietnamese home garden accessions clustered together in the STRUCTURE analyses. Based on the PCoA, they could be further divided into two genetic groups. A first group (Fig. 2a, pink circle) mainly consisted of accessions from the Philippines and one accession from Flores, Indonesia. The accession ITC0248 “Singapuri” was genetically identical to ITC1588 “Lal Velchi” from India and both accessions clustered in this first group (Fig. 3). In the second group, (Fig. 2a, yellow circle), almost all ITC accessions from multiple countries of origin were genetically identical for the studied markers. This group also included six samples from Vietnamese home gardens, suggesting these ITC accessions were likely sampled from cultivated (potentially Vietnamese) rather than wild material and later on distributed to different countries.

Based on our results, we cannot confirm whether Philippine *M. balbisiana* accessions can be considered cultivated, as proposed by de Langhe et al. (2015), as the genetic distinctness of these accessions from wild populations of China and Vietnam could represent mainland-island differentiation within the natural distribution of the species (Franks 2010). Recently, high phenotypic diversity was found in 97 M*. balbisiana* accessions from the ex situ collection of the National Plant Genetic Resources Laboratory in Los Baños (Philippines) and the conservation gaps of this collection were highlighted (Sotto and Rabara 2000; Rabara et al. 2020). High genetic diversity was also reported in *M. balbisiana* in the Philippines, but only cultivars from multiple Philippine collections and genomic groups were considered in their study (Doloiras-Laraño et al. 2018). Linguistic evidence exists that *M. balbisiana* was likely translocated southwards from South China following a trail to New Guinea over the Philippines. This theory is further supported based on triploids with a component from *M. acuminata* subsp. *banksii*, which is not found in more northern parts of the Philippines (Perrier et al. 2009, 2011). Thousands of years of use and semi-cultivation of *M. balbisiana* might explain why it is widespread in the Philippines and may be the cause of relatively high genetic diversity (De Langhe et al. 2015). Our results provide evidence for a close relationship of Philippine ITC accessions with some individuals from Chinese populations (Fig. 2b) and especially from South China and West China, though individuals were assigned to different clusters. Additional research with more exhaustive sampling is needed to further investigate the relationship between (southern) Chinese and Philippine *M. balbisiana* accessions.

Though passport data of “Lal Velchi” (ITC1588) were missing, its origin was assessed as a wild accession from India (pers comm. Van den houwe). However, its genetic differentiation from other populations sampled from the native distribution area of mainland Asia and its high genetic similarity to ITC0248 “Singapuri” suggests that this accession might also have been translocated and domesticated in other regions. Suckers of this genotype may have been distributed between villages, also in more southern states and Sri Lanka (Uma 2006). Additional screening of genetic diversity of wild *M. balbisiana* populations from India is critical, especially because India is considered as a secondary centre of hybridisation of native *M. balbisiana* with diploid *M. acuminata* cultivars after their import from Maritime Southeast Asia (Simmonds and Shepherd 1955; Uma 2006; Perrier et al. 2009). Moreover, recent phytolith evidence suggested Sri Lanka might also have been a centre for early banana dispersal and that exchange of banana cultivars between India and Sri Lanka might already have taken place through maritime network connections in the middle of the fifth millennium before present (Premathilake and Hunt 2018).

The second genetic group (Fig. 2, yellow circle) contained five ITC accessions as well as the home garden samples from Vietnam. Strikingly, 7 out of 11 accessions were genetically identical based on the assessed markers. this includes most home garden samples as well as both Vietnamese ITC accessions, ITC1587 “Pisang Klutuk Wulung” from Indonesia, ITC1787 from the Philippines, and ITC1120 “Tani” from Thailand. Because the accession from Thailand is not georeferenced and little passport data are available, we assume that it was not sampled from a native population in Thailand but rather from cultivated material imported from elsewhere. To our knowledge, no real wild populations of *M. balbisiana* have been reported from northern Thailand. De Langhe et al. (2000) and Simmonds (1956) suggested a human introduction and cultivation of *M. balbisiana* in evergreen forests of northern Thailand (Nan province), even if populations seem to appear in natural conditions and suitable climate. Similarly, “Pisang Klutuk Wulung” (ITC1587), and *M. balbisiana* in general, was likely introduced to Java and naturalised there (Simmonds 1956; De Langhe et al. 2015). Based on the patterns of genetic structure, *M. balbisiana* plants from home gardens in Vietnam were most likely introduced from germplasm accessions or clones from e.g. the genetically similar accessions “Pisang Klutuk Wulung” or “Tani”. Other studies using different markers also showed the genetic relatedness of “Tani” and “Pisang Klutuk Wulung” accessions among other accessions. For accession VNM-S7, according to locals, *M. balbisiana* was imported from the North after the Vietnam war because it is rather drought resistant and used for feed, typically for cows, though no genetic link with a native population could be found (pers. comm.). This suggests that *M. balbisiana* used in home gardens might have come from cultivated material rather than from wild populations (Duroy et al. 2016; Jeensae et al. 2021). In contrast to other Philippine accessions, the clustering of ITC1787 with this group and the genetic uniformity with the other accessions within this group suggests that clones were likely shared between different locations, e.g. as in vitro material or as suckers. A similar study in the Democratic Republic of the Congo compared the gene pool of wild populations of Robusta coffee (*Coffea canephora* Pierre ex A.Froehner) with cultivated accessions from the Institut National des Etudes et Recherches Agronomique (INERA) Yangambi and from local backyards (Vanden Abeele et al. 2021). *Coffea canephora* plants grown in home gardens were likely directly or indirectly received from INERA breeding programmes and both the accessions from INERA and from backyards were genetically distinct from the local wild gene pool. Hence, it seems that, at least for some crops, planting material from elite cultivars that were bred elsewhere is preferred in backyards above semi-cultivated accessions related to the local gene pool.

The eight ITC accessions for which the country of origin was not known showed additional genetic diversity of *M. balbisiana* compared to the accessions with a known country of origin, possibly representing populations in a different part of the species’ distribution area. These findings again stress the need for passport data in order to more efficiently collect additional germplasm. Three of those accessions showed genetic similarity to accessions from West China, while one (ITC0271, “Eti Kehel”) showed genetic similarity with some samples from China and two ITC accessions from the Philippines. As *M. balbisiana* is locally known as Eti-Kehel in Sri Lanka and as ITC0271 was donated to ITC by the Royal Botanic Gardens of Sri Lanka, Sri Lankan origin of this accession can be assumed (Liyanage et al. 1998; Duroy et al. 2015). ITC0271 was genetically most similar to a group containing three Philippine accessions and the accessions ITC1588 “Lal Velchi” from India and ITC0248 “Singapuri”, providing some evidence for the exchange of genetic material between India, the Philippines, and Singapore. Due to the limited number of accessions from the Philippines, India, or other countries of origin apart from China and Vietnam, the country of origin for the other ITC accessions could not be inferred.

#### Papua New Guinea

In 1956, Simmonds described the appearance of *M. balbisiana* in New Guinea as widespread and locally abundant in natural habitats, suggesting it could be truly native (Simmonds 1956). Since then, however, it was proposed that PNG does not belong to the native distribution area (Argent 1976; De Langhe et al. 2015). We showed that samples from PNG, both from the seed collection and ITC, were genetically distinct from the other clusters. The very low number of polymorphic loci (0% for both ITC accessions and 11% for the seed collection) further support the hypothesis that *M. balbisiana* was introduced to PNG thousands of years ago (before ca 3100 years), represented by only one or very few genotypes or “BB” cultivars (Argent 1976). The relatively large genetic differentiation *between M. balbisiana* accessions from PNG and the Philippines that was observed in this study does not support a dispersal of wild *M. balbisiana* from south China to PNG over the Philippines. Hence, a wild origin of *M. balbisiana* in PNG via this dispersal route seems to be less likely.

### The value of ITC in distributing *Musa balbisiana* diversity

Among the 6,772 Musaceae accessions from multiple germplasm collections around the world listed on the Taxonomy Browser of the MGIS, *M. balbisiana* is represented by 167 accessions (as of May 2021). Of these, 145 are labelled as “wild species or subspecies” (Ruas et al. 2017). Very little passport data are available and only 28 accessions were available for distribution from the ITC and were therefore included in this study. Based on our SSR data analysis we can conclude that most of the *M. balbisiana* accessions held at ITC were genetically distant to the accessions that were recently collected in the native distribution range of the species (Janssens et al. 2016). Only one accession (ITC1527) sampled from Xishuangbanna in Southwest China grouped with samples of Vietnam and neighbouring Chinese provinces. All other accessions obtained from different countries in Asia and PNG were systematically assigned to separate clusters. This indicates on the one hand that the ITC conserves and distributes unique and valuable genetic diversity of *M. balbisiana* that is likely not present in the seed collections sampled from Vietnamese populations that are held at the Millennium Seed Bank (MSB) or from seed collections from China. These include Vietnamese seed collections of populations of which leaves were used in this study. On the other hand, very limited germplasm from other countries of the native distribution area of *M. balbisiana* is available at the ITC or at other germplasm centres, though these populations have relatively high genetic variation within- and among-populations compared to feral populations and cultivated material (Ge et al. 2005; Bawin et al. 2019; Mertens et al. 2021a).

In addition to the one ITC accession from China, nine other ITC accessions originated from a country in which *M. balbisiana* is assumed to be native. However, the wild origin of these nine ITC accessions is uncertain. Two of those accessions were georeferenced to the Nghe An province of Vietnam (ITC1681 and ITC1687), the same province in which the populations VNM-C1 and VNM-C2 were sampled. Based on the provided coordinates, these ITC accessions were sampled from more densely populated areas in the East of the province in contrast to the populations which were collected in the West, potentially from home gardens instead of a wild population. Two other accessions (ITC1120 and ITC1588) were sampled from Thailand and India, respectively. Both countries are partly covered by the (sub)tropical forests of northern Indo-Burma, which harbour many wild *Musa* populations (Janssens et al. 2016; Mertens et al. 2021b). While these accessions have been used multiple times in previous studies as representatives of the wild diploid “BB” genomic group, their wild origin remains uncertain due to the lack of passport data (Ruas et al. 2017; Christelová et al. 2017; Zuo et al. 2018; Nakato et al. 2019; Igwe et al. 2021). The five remaining ITC accessionswere of Philippine originand showed relatively high genetic variation. Ongoing genetic screening of wild *M. balbisiana* at the University of the Philippines Los Baños also suggests high diversity in the Philippines (Gueco, pers. comm.). This high genetic diversity in Philippine *M. balbisiana* may suggest that the Philippines are part of the species’ native distribution area. Nevertheless, it remains unclear whether this high diversity reflects naturally accumulated genetic variation in wild *M. balbisiana* populations or increased variation in feral populations. Such increased variation in feral populations can be explained by multiple introductions of *M. balbisiana* into the Philippines or introgression of genetic material from other *Musa* species (e.g. *M. acuminata*) into the Philippine *M. balbisiana* gene pool (Hufford et al. 2019). Chloroplast sequences with high sequence similarity to *M. acuminata* of three Philippine ITC *M. balbisiana* accessions (ITC1788, ITC1789, ITC1823) provide some support that introgression could have taken place.

The lack of sufficient germplasm from the putative regions of origin of this species results in a poor representation of the total genetic diversity of the species at ITC, especially with only one sample from India and Thailand and none from Laos and Myanmar, all countries of which at least some parts are climatically suitable for the species (Janssens et al. 2016; Mertens et al. 2021b; POWO 2022). Current germplasm accessions should be maintained and, where available, passport data should be updated. Because of the limited knowledge on the genetic diversity of *M. balbisiana* populations in countries such as Myanmar, Thailand, India, Laos, but also the Philippines and Indonesia, additional collecting and genetic screening of populations and existing collections (such as the MSB) with plant material from these countries is required to maximize the genetic representation of the species with minimal resources (e.g. with seed collections).

### Optimisation of banana germplasm sampling for conservation and breeding purposes

Based on our findings, there is an urgent need for new and additional sampling of *Musa balbisiana* germplasm, especially from countries where the species is native. For *M. balbisiana* specifically, despite its higher resistance to biotic and abiotic stresses compared to *M. acuminata* (Nelson et al. 2006; Ocan et al. 2008; Vanhove et al. 2012; Mattos-Moreira et al. 2018; Tripathi et al. 2019b), the largest drawback in using this species for breeding is the presence of endogenous banana streak virus (eBSV) in the genome of genotypes containing at least one “B” genome as progenitor. This results in spontaneous infections of the hybrids following abiotic stress, making it a major constraint for use in breeding programmes. However, marker-assisted breeding, the discovery of *M. balbisiana* plants that contain non-infective eBSV sequences, and recent advances in gene-editing technology are promising to support a more efficient use of *M. balbisiana* genetic resources (Duroy et al. 2016; Umber et al. 2016; Tripathi et al. 2019a).

The mating system of wild banana species is an important indicator to optimise seed sampling strategies. For (partially) cross-pollinated species such as *Musa balbisiana*, increasing the number of sampled populations is more effective in maximizing genetic capture than sampling more mother plants per population (Kallow et al. 2021) though within-species variation in mating system should be taken into account. To further maximize genetic capture, large populations distant from anthropogenic activities should be prioritised (Andersson and de Vicente 2010; Almeida-Rocha et al. 2020; Kallow et al. 2021).

Equally important is making sure passport data of germplasm collections are of high quality for optimal use for farmers or breeders, but also that the material is available for research. Optimally, passport data follow a standard of descriptors such as the standard of the multi-crop passport descriptors from FAO and Bioversity International (Alercia et al. 2015). Such data typically include information on the collection and the storage of the germplasm accession itself but should also include a detailed description of the geographical origin and environmental conditions of the sampled location. The status of the accession (wild, semi-wild, cultivated or cultivar) should additionally be recorded as well as phenotypic, morphological, and agronomic traits. When this information is consistently provided for accessions in germplasm collections, their use for research (e.g. for determining conservation gaps) or breeding purposes can more easily be evaluated (Weise et al. 2020).

For bananas, additional information might further promote the collection and use of specific germplasm accessions related to pest management and desirable traits. It is known that the economically most devastating fungal pathogen of banana*,* the soil borne fungus *Fusarium oxysporum* f.sp. *cubense* (*Foc*)*,* often is symptomless but present in the field which could lead to the spread of *Fusarium* when locally distributed. Screening wild genotypes of *M. balbisiana* for resistance against *Foc* might help in prioritising areas for additional germplasm collection. For example, though five Indian BB-type accessions were found highly resistant or immune to *Foc* race 1 (Thangavelu et al. 2021), germplasm of Indian *M. balbisiana* is largely missing at the ITC. Investigating or sampling the root microbiome might give information on whether the fungus is present. Recent studies highlighted a significant change in endophytic microbial and fungal community composition during disease development compared to non-symptomatic plants (Kaushal et al. 2020a, b). Passport data related to the root microbiome could therefore also be important for selecting genetic material for breeding programmes.

## Conclusions

Until now, little of the wild *M. balbisiana* genetic diversity from the native distribution area is captured at the ITC and thus available for distribution and research. By investigating both wild populations as well as germplasm accessions from multiple countries of origin held at the ITC, we found that passport data are often missing and incomplete. The country of origin was unknown for eight out of 28 accessions and only the country was supposedly known for an additional 11 accessions, making it more difficult to evaluate an accession as wild material on the one hand and limiting its use for breeding on the other hand. While considerable genetic variation is found in accessions from the Philippines and wild populations from China and Vietnam, it is clear that *M. balbisiana* and the genus *Musa* in general is a very complex group due to multiple and repeated events of migration and intensive cultivation of the species during the last millennia. Assessing the genetic structure revealed that samples could be systematically subdivided into three to six genetic groups, with a clear separation of most ITC accessions and samples from home gardens, samples from PNG, and populations sampled in China and Vietnam. Because most of the distributed material of *M. balbisiana* from the ITC is genetically similar, we here suggest that more germplasm should be collected from wild populations in China and Vietnam, but especially also in northeastern India, Myanmar, and the Philippines and that existing collections in the world should be genetically screened. High throughput sequencing techniques are necessary to further explore to what extent Philippine accessions can be considered as wild or whether they are more likely introduced from South China for cultivation.

## Supplementary Information

Below is the link to the electronic supplementary material.

Supplementary file1 (DOCX 331 kb)

Supplementary file2 (DOCX 35 kb)

## Data Availability

Sanger sequences generated in this project were submitted to GenBank (Accession numbers OK648712–OK649230).
