# Supplementary material for: Phylogeography and conservation gaps of Musa balbisiana Colla genetic diversity revealed by microsatellite markers
Source: Genet Resour Crop Evol. 2022 May 7;69(7):2515–34. doi: 10.1007/s10722-022-01389-4 (PMC9393128; doi:10.1007/s10722-022-01389-4)
Supplement: Supplementary file 1 — Supplementary file1 (DOCX 331 kb) [file 10722_2022_1389_MOESM1_ESM.docx]

**Genetic Resources and Crop Evolution**

**Phylogeography and conservation gaps of *Musa balbisiana* genetic diversity revealed by microsatellite markers**

Arne Mertens^1,2*^, Yves Bawin^2,3^, Samuel Vanden Abeele^2^, Simon Kallow^1,4^, Rony Swennen^1,5^, Dang Toan Vu^6^, Tuong Dang Vu^6^, Ho Thi Minh^6^, Bart Panis^7^, Filip Vandelook^2^, Steven B. Janssens^2,3^

^1^Department of Biosystems, Laboratory of Tropical Crop Improvement, KU Leuven, Leuven, Belgium

^2^Meise Botanic Garden, Meise, Belgium

^3^Department of Biology, KU Leuven, Leuven, Belgium

^4^Royal Botanic Gardens Kew, Millennium Seed Bank, Ardingly, United Kingdom

^5^International Institute of Tropical Agriculture, Kampala, Uganda

^6^Research Planning and International Department, Plant Resources Center, VAAS, Hanoi, Vietnam

^7^Bioversity International, Leuven, Belgium

*Corresponding author

E-mail: [arne.mertens70@gmail.com](mailto:arne.mertens70@gmail.com)


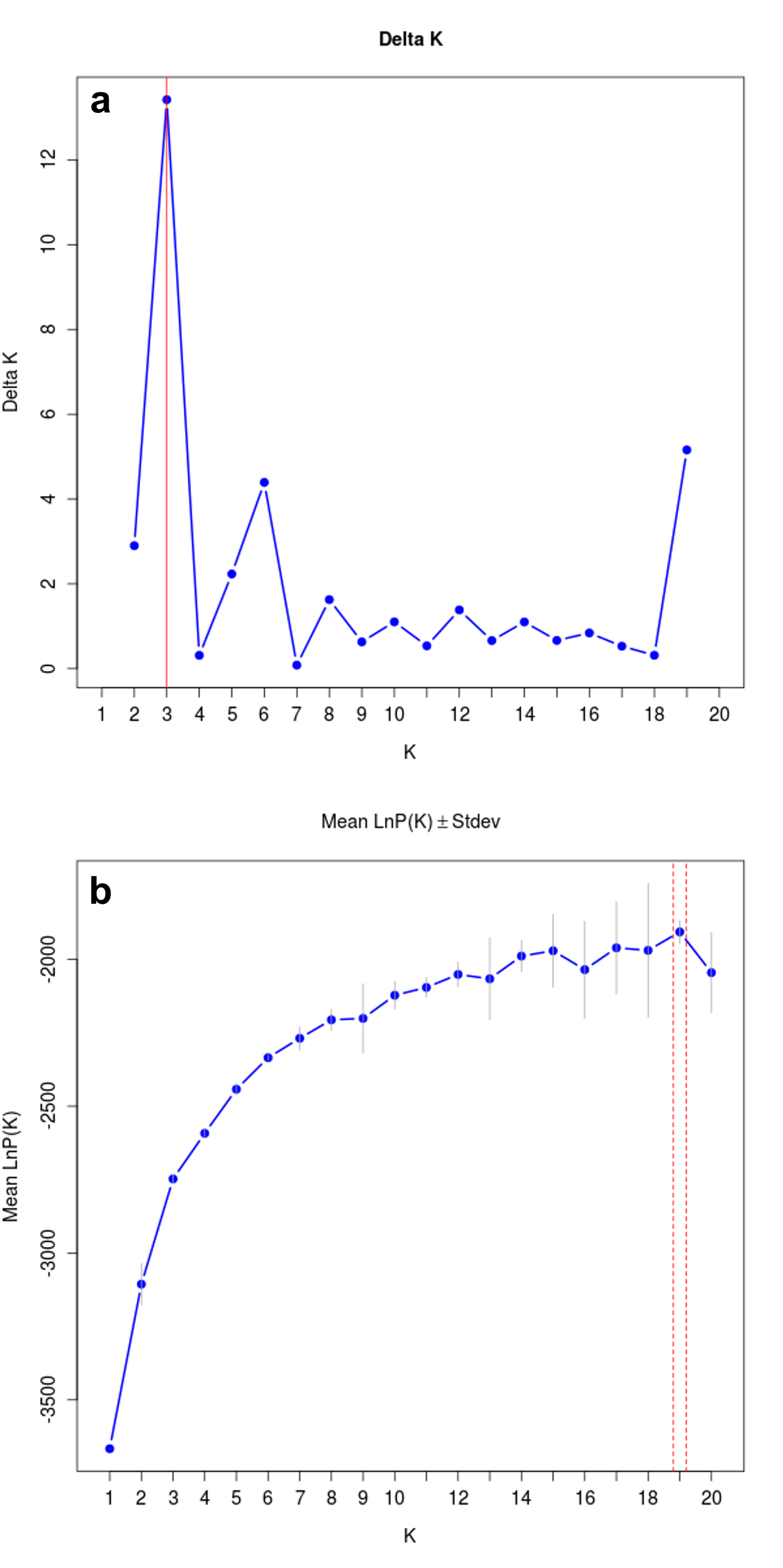


**Supplementary Figure 1.** Determination of the optimal number of clusters (K) in the complete Musa balbisiana microsatellite dataset. **a** Evanno method ΔK/K. **b** Mean LnP(K) / Mean LnP(K). The optimal K is marked with a red line in both figures (Evanno et al., 2005)

**References**

Evanno, G., Regnaut, S., Goudet, J., 2005. Detecting the number of clusters of individuals using the software STRUCTURE: A simulation study. Mol. Ecol. 14, 2611–2620. https://doi.org/10.1111/j.1365-294X.2005.02553.x
