# Supplementary material for: Phylogeography and conservation gaps of Musa balbisiana Colla genetic diversity revealed by microsatellite markers
Source: Genet Resour Crop Evol. 2022 May 7;69(7):2515–34. doi: 10.1007/s10722-022-01389-4 (PMC9393128; doi:10.1007/s10722-022-01389-4)
Supplement: Supplementary file 2 — Supplementary file2 (DOCX 35 kb) [file 10722_2022_1389_MOESM2_ESM.docx]

**Genetic Resources and Crop Evolution**

**Phylogeography and conservation gaps of *Musa balbisiana* genetic diversity revealed by microsatellite markers**

Arne Mertens^1,2*^, Yves Bawin^2,3^, Samuel Vanden Abeele^2^, Simon Kallow^1,4^, Rony Swennen^1,5^, Dang Toan Vu^6^, Tuong Dang Vu^6^, Ho Thi Minh^6^, Bart Panis^7^, Filip Vandelook^2^, Steven B. Janssens^2,3^

^1^Department of Biosystems, Laboratory of Tropical Crop Improvement, KU Leuven, Leuven, Belgium

^2^Meise Botanic Garden, Meise, Belgium

^3^Department of Biology, KU Leuven, Leuven, Belgium

^4^Royal Botanic Gardens Kew, Millennium Seed Bank, Ardingly, United Kingdom

^5^International Institute of Tropical Agriculture, Kampala, Uganda

^6^Research Planning and International Department, Plant Resources Center, VAAS, Hanoi, Vietnam

^7^Bioversity International, Leuven, Belgium

*Corresponding author

E-mail: [arne.mertens70@gmail.com](mailto:arne.mertens70@gmail.com)

**Supplementary Table 1** Musa balbisiana genetic material used in this study. Country of origin, population/accession code, sample size, locality and geographical coordinates are given where available. In vitro samples retrieved from the ITC are referenced with their respective ITC accession number. Material source and status are presented for all populations or accessions

| Country | Code | Sample size | Locality | Latitude | Longitude | Source | Status |
| --- | --- | --- | --- | --- | --- | --- | --- |
| Vietnam | VNM-N1 | 16 | Lao Cai, Muong Cau | 22.314 | 104.040 | Mertens et al. (2021a) | Leaf/wild |
| Vietnam | VNM-N2 | 15 | Lai Chau, Can Cau | 22.437 | 103.450 | Mertens et al. (2021a) | Leaf/wild |
| Vietnam | VNM-N3 | 11 | Lai Chau, Cu Ti | 22.327 | 103.486 | Mertens et al. (2021a) | Leaf/wild |
| Vietnam | VNM-N4 | 15 | Lai Chau, Na Bo | 22.345 | 103.518 | Mertens et al. (2021a) | Leaf/wild |
| Vietnam | VNM-N5 | 13 | Lai Chau, Nam Ma Dao | 22.408 | 103.316 | Mertens et al. (2021a) | Leaf/wild |
| Vietnam | VNM-N6 | 13 | Lai Chau, Seo Leng | 22.445 | 103.261 | Mertens et al. (2021a) | Leaf/wild |
| Vietnam | VNM-N7 | 13 | Bao Thang, Suoi Thau | 22.298 | 104.042 | Mertens et al. (2021a) | Leaf/wild |
| Vietnam | VNM-N8 | 11 | Van Ban, Khanh Yen Thuong | 22.115 | 104.244 | Mertens et al. (2021a) | Leaf/wild |
| Vietnam | VNM-N9 | 15 | Van Ban, Nam Ma | 22.198 | 104.184 | This study | Leaf/home garden |
| Vietnam | VNM-N10 | 15 | Dien Bien, Xa Tu | 21.450 | 103.440 | Mertens et al. (2021a) | Leaf/wild |
| Vietnam | VNM-N11 | 14 | Dien Bien, Hin 1 | 21.697 | 103.111 | Mertens et al. (2021a) | Leaf/wild |
| Vietnam | VNM-N12 | 15 | Lai Chau, Huoi Danh | 22.142 | 102.973 | Mertens et al. (2021a) | Leaf/wild |
| Vietnam | VNM-N13 | 15 | Lai Chau, Huoi Han | 22.387 | 102.781 | Mertens et al. (2021a) | Leaf/wild |
| Vietnam | VNM-N14 | 4 | Dien Bien, Xa Tu | 22.550 | 103.297 | Mertens et al. (2021a) | Leaf/wild |
| Vietnam | VNM-C1 | 15 | Nghe An, Ban Phung | 19.299 | 104.321 | Mertens et al. (2021a) | Leaf/wild |
| Vietnam | VNM-C2 | 15 | Nghe An, Khe Ngau | 19.303 | 104.385 | Mertens et al. (2021a) | Leaf/wild |
| Vietnam | VNM-S1 | 15 | Kontum, Mo Rai | 14.356 | 107.662 | Mertens et al. (2021a) | Leaf/wild |
| Vietnam | VNM-S2 | 10 | Kontum, Dak Nhoong | 15.081 | 107.682 | Mertens et al. (2021a) | Leaf/wild |
| Vietnam | VNM-S3 | 1 | Dak Lak, Krong Na | 12.292 | 108.292 | This study | Leaf/home garden |
| Vietnam | VNM-S5 | 1 | Dak Lak, Ea Trul | 12.508 | 108.267 | This study | Leaf/home garden |
| Vietnam | VNM-S6 | 1 | Dak Lak, Ea Trul | 12.508 | 108.267 | This study | Leaf/home garden |
| Vietnam | VNM-S7 | 1 | Dak Lak, Ea Dar | 12.820 | 108.524 | This study | Leaf/home garden |
| Vietnam | VNM-S8 | 1 | Dak Lak, Ea Sar | 12.905 | 108.469 | This study | Leaf/home garden |
| Vietnam | VNM-S9 | 1 | Gia Lai, Chu Se | 13.639 | 108.109 | This study | Leaf/home garden |
| China | CHN-W1 | 12 | Yunnan | 24.700 | 97.567 | Bawin et al. (2019) | Seed/wild |
| China | CHN-W2 | 14 | Yunnan | 24.700 | 97.567 | Bawin et al. (2019) | Seed/wild |
| China | CHN-W3 | 14 | Yunnan | 24.700 | 97.567 | Mertens et al. (2021a) | Seed/wild |
| China | CHN-W4 | 8 | Yunnan | 24.700 | 97.567 | Mertens et al. (2021a) | Seed/wild |
| China | CHN-S | 5 | Guangdong | / | / | Mertens et al. (2021a) | Seed/wild |
| China | CHN-Hainan | 15 | Hainan | 19.517 | 109.483 | Bawin et al. (2019) | Seed/wild |
| Japan | JPN-Amami | 15 | Amami | 28.133 | 129.333 | Bawin et al. (2019) | Seed/introduced |
| Unknown | ITC0080 | 1 | unknown | / | / | This study | Field collection/ 2x |
| Unknown | ITC0094 “Balbisiana (10852)” | 1 | unknown | / | / | This study | Field collection/ 2x |
| Unknown | ITC0211 | 1 | unknown | / | / | This study | Field collection/ 2x |
| Unknown | ITC0212 | 1 | unknown | / | / | This study | 2x |
| Unknown | ITC0246 “Cameroun” | 1 | unknown | / | / | This study | Field collection/ 2x |
| Unknown | ITC0247 “Honduras” | 1 | unknown | / | / | This study | Field collection/ 2x |
| Unknown | ITC0248 “Singapuri” | 1 | unknown – Singapore? | / | / | This study | Field collection/ 2x |
| Sri Lanka | ITC0271  “Eti Kehel” | 1 | unknown – Sri Lanka? | / | / | This study | Botanic Garden/ 2x |
| Philippines | ITC0342 | 1 | Philippines | / | / | This study | Field collection/ 4x |
| Unknown | ITC0545 | 1 | unknown | / | / | This study | Botanic garden/ 2x |
| Philippines | ITC0564 “Butuhan, salient apex” | 1 | Philippines | / | / | This study | Unknown/ 2x |
| Philippines | ITC0565 “Butuhan, intermediate apex” | 1 | Philippines | / | / | This study | Unknown/ 2x |
| Papua New Guinea | ITC0626 | 1 | New Britain | -4.350 | 152.271 | This study | 2x |
| Papua New Guinea | ITC1016 | 1 | Oro province | -8.917 | 148.400 | This study | 2x |
| Thailand | ITC1120 “Tani” | 1 | Thailand | / | / | This study | Germplasm collection/2x |
| China | ITC1527 | 1 | Xishuangbanna | 22.019 | 100.914 | This study | Botanic garden/ 2x |
| Indonesia | ITC1587 “Pisang Klutuk Wulung” | 1 | Indonesia | / | / | This study | Field collection/ 2x |
| India | ITC1588 “Lal Velchi” | 1 | India | / | / | This study | Field collection/ 2x |
| Vietnam | ITC1681 “Chuoi hot” | 1 | Nghe An, Dong son | 18.917 | 105.317 | This study | Field collection/ 2x |
| Vietnam | ITC1687 “Chuoi hot qua lep” | 1 | Nghe An, Dong son | 18.917 | 105.317 | This study | Field collection/ 2x |
| Philippines | ITC1780 | 1 | Sorsegon | / | / | This study | Field collection/ 2x |
| Philippines | ITC1787 “Musa balbisiana 99-058” | 1 | Leyte | / | / | This study | Field collection/ 2x |
| Philippines | ITC1788 “Musa balbisiana 99-088” | 1 | Philippines | / | / | This study | Field collection/ unknown |
| Philippines | ITC1789 “Musa balbisiana 98-364” | 1 | Philippines | / | / | This study | Field collection/ unknown |
| Philippines | ITC1790 “Musa balbisiana 98-573” | 1 | Philippines | / | / | This study | Field collection/ unknown |
| Philippines | ITC1823 “Musa balbisiana 98-633” | 1 | Philippines | / | / | This study | Field collection/ 2x |
| Japan | ITC1850 “Musa balbisiana var.liukiuensis” | 1 | Amami | / | / | This study | Botanic garden/ 3x |
| Indonesia | ITC1875 “Mu'u Popot” | 1 | Flores | / | / | This study | Field collection/ unknown |
| Papua New Guinea | PNG | 15 | Morobe | -6.683 | 146.933 | Bawin et al. (2019) | Seed/wild? |
